# Predominant polarity as a potential moderator of group CBT outcomes in bipolar disorder: an exploratory *post-hoc* analysis

**DOI:** 10.3389/fpsyt.2026.1697134

**Published:** 2026-03-09

**Authors:** Tatiana Cohab, Gabriel Okawa Belizário, Beny Lafer, Bernardo Carramão Gomes

**Affiliations:** 1 Bipolar Disorder Program (PROMAN), Department & Institute of Psychiatry, University of São Paulo Medical School, São Paulo, Brazil; 2 Department of Psychiatry, Universidade Federal de São Paulo, São Paulo, Brazil

**Keywords:** bipolar disorder, cognitive behavioral therapy, group psychotherapy, moderator, predominant polarity

## Abstract

**Introduction:**

Bipolar disorder (BD) is a chronic and severe mood disorder, characterized by recurrent episodes of mania, hypomania, and depression. Adjacent to pharmacotherapy, positive evidence has been found for the benefit of adding cognitive behavioral therapy (CBT), family-focused therapy (FFT), interpersonal and social-rhythm therapy (IPSRT) and Group Psychoeducation for treating BD.

**Objective:**

CBT is well established as a form of psychotherapy for bipolar disorders, although variables that may affect its results are still poorly studied. The present study aimed to investigate Predominant Polarity (PP) as a possible moderator of response to group CBT for BD.

**Methods:**

The original controlled study included 50 bipolar patients, divided into two groups: one was maintained on treatment as usual (TAU) solely, and a second group was assigned to 18 weekly group CBT sessions as an add-on treatment to TAU. A 16-month follow-up was conducted after the sessions, as patients were evaluated by phone by two psychiatrists, blind to the patients’ condition. We have conducted a *post-hoc* analysis including solely individuals assigned to group CBT, dividing patients according to predominant manic or depressive polarity.

**Results:**

Log-rank survival analysis revealed manic predominant polarity patients as maintaining an episode-free status for a longer amount of time in comparison to depressive predominant polarity patients.

**Conclusion:**

The results suggest predominant polarity may be an important moderator of CBT response in BD. Further studies should include larger samples.

## Introduction

Bipolar disorder (BD) is a chronic and severe mood disorder, characterized by recurrent episodes of mania, hypomania, and depression (1). It currently affects more than 1% of the world’s population, and mortality rates are high among those affected, especially death by suicide (1, 2). No biomarkers have yet been found for this disorder, which makes the DSM-5 and CID-10 the main guides for diagnosis (3). Due to high rates of relapse present in this disorder, targeting only acute mood episodes is insufficient; both pharmacological and psychological interventions are required in order to prevent future episodes (1). Furthermore, a considerable percentage of BD patients do not respond adequately to the best available pharmacological treatment (4), reinforcing the need for new effective treatments following what has been done in conditions such as obsessive compulsive disorder for instance (5–7). It should be noted that the adequate treatment of comorbidity between bipolar disorder and other conditions, whether through psychotherapeutic (8) or pharmacological approaches (9), remains a significant clinical challenge.

As an add-on condition, positive evidence has been found for cognitive behavioral therapy (CBT), family-focused therapy (FFT), interpersonal and social rhythm therapy (IPSRT), and group psychoeducation (10). Originally designed to treat unipolar depression patients, CBT is currently the psychotherapeutic approach with the most tested efficacy to treat various psychiatric disorders (11). Regarding BD, CBT aims to educate patients and family members about the disorder, encourage patients to have an active role in the treatment, monitor and foresee symptom appearances, and deal with dysfunctional thoughts (12, 13). The majority of CBT protocols for BD are applied during euthymia and target relapse prevention and symptomatology reduction (13, 14). Recent meta-analysis suggests that CBT is less effective than previously expected in BD, especially regarding preventing new mood episodes (13). A possible explanation for this is the high heterogeneity in clinical presentation of BD, with emerging new questions about which patients could benefit from different psychological approaches available (13).

The diagnosis of BD is oftentimes accompanied by specifiers as a means to predict the course of the disorder and tools for treatment (15). Recently, studies have suggested Predominant Polarity (PP) as a meaningful course specifier for the disorder, branching patients into distinct groups and providing another means for personalizing their treatment (16–18). PP can be divided into three distinct categories: Manic Predominant Polarity (MPP), Depressive Predominant Polarity (DPP) and Indefinite Predominant Polarity (IPP) (17). Nonetheless, definitions for the specifiers remain divided between requiring patients to have at least 2/3 of all episodes in one polarity (17) and requiring patients to have more than 1/2 of episodes in one polarity (19). MPP was associated with earlier age of onset, male gender, BD Type 1 and presence of psychotic symptomatology (20). In addition to being associated with a greater number of depressive episodes and a greater total number of episodes throughout life, PPD is more common in women and is related to a greater history of suicide attempts (21). PP also appears to correlate with differences in cognitive impairment commonly reported in BD. One recent study has compared the cognitive performance in DPP, IPP, and MPP and healthy controls (22). In summary, in comparison to HC, euthymic MPP performed worse in processing speed, problem-solving, visual learning and memory, verbal learning and memory, working memory, attention, and global cognition. In this study, DPP group presented the least cognitive impairment, followed by the IPP group.

Although CBT is well established as a form of psychotherapy for several psychiatric disorders, variables that may affect its results are still poorly studied for BD. Scott et al. (23) in a *post-hoc* analyses found that the number of previous mood episodes was a determining factor in preventing new episodes in patients who underwent individual CBT as add-on TAU treatment. Some studies have failed in the identification of moderators of response in psychotherapy for BD (24). However, the same did not occur in the cognitive rehabilitation literature, since some authors found a potential association between the perception of cognitive impairment and the response to the rehabilitation program (25). Considering the high variability to psychotherapy response in BD found in meta-analyses (13), it is essential to identify potential response moderators in order to better personalize the treatment of these individuals.

The present study is a secondary *post-hoc* analysis of a previously published randomized controlled trial of Cognitive Behavioral Group Therapy (CBGT) with euthymic BD individuals (see 26). Our group originally opted for the group therapy format due to the high demand for mental health care in the Brazilian public health system. Cognitive group therapy has good evidence for treating conditions such as schizophrenia (27), comparable to or even superior to individual treatment. Group approaches had already been successfully tested in BD patients, mainly increasing the time to remission and reducing mood symptoms (28). This may be due to unique elements observed in the group format, such as the exchange of experiences among patients and the collective training of social skills, what has been observed in BD and in several other medical conditions (29). We hypothesized that distinct PP groups would exhibit differential responses to CBGT.

## Method

All participants were outpatients at the Institute of Psychiatry (Bipolar Disorder Research Program, PROMAN), University of São Paulo Medical School, São Paulo, Brazil. The following were the inclusion criteria: a DSM-IV diagnosis of BD type I or II based on the Structured Clinical Interview for DSM-IV (SCID IV) (30), an age between 18 and 60 years, euthymic state (Young Mania Rating Scale score <6 and 17-item Hamilton Depression Rating Scale score <8) at the time of enrollment in the study, >5 years of schooling, and the use of at least one mood stabilizer or atypical antipsychotic medication. Exclusion criteria were substance use disorders in the last six months and organic brain disorder. Predominant polarity was defined following the proposal of the Barcelona Group, that is, at least 2/3 of episodes throughout life for mania, hypomania, or depression (19). This study has been approved by the local ethical committee (CaPPesq, protocol number: 0261/07).

After initial assessment, all patients were randomized into two groups: the treatment as usual group (TAU), in which patients received only pharmacological treatment during individual medical consultations, and the CBGT group, in which CBGT was adjunctive to pharmacological treatment (TAU) (see 26). Therapists conducting the groups were formally trained in CBT and had over five years of experience. All patients received the same written material on CBT for BD in a manual especially developed for this study. The length of the study was twenty-two weeks of the initial phase + two years of follow-up.

This article is a secondary *post-hoc* analysis including only the 22 euthymic bipolar patients originally assigned to 18 weekly CGBT sessions and have completed one year of following up (26). The first follow-up assessment was obtained six months after the beginning of the first group therapy (post-treatment). The SCID mood module for the past six months and standardized questions about relapses, time to recovery and suicide attempts were administered by two experienced psychiatrists, who were blinded to the participant’s group. This standard interview was applied again every three months after that stage. All follow-up assessments were conducted by telephone to ensure adequate frequency and avoid difficulties with patient mobility, especially during depressive episodes. Time to relapse was defined as the number of weeks until a new DSM-IV mood episode occurred and all individuals included in the CBGT needed to complete at least 9 of the 18 sessions of the protocol (50%).

Predominant Polarity (PP) was defined as having a majority of past lifetime episodes of a specific polarity (16); patients presenting a majority of past depressive episodes were allocated to the depressive predominant polarity (DPP) while patients presenting a majority of manic episodes were allocated to the manic predominant polarity (MPP) group. Demographic and clinical variables were compared between groups through Student’s t-test or Mann-Whitney U-tests, depending on the normality of the variables, as assessed by a Kolmogorov-Smirnov test. A log-rank survival analysis (Kaplan-Meyer) was employed to assess differences in time to the first episode between the groups. For all these measures, we assumed a significance level alpha of 5%.

A sample size calculation was conducted based on a five-year study evaluating the role of PP on prospective course of BD (31). Assuming that 23% of participants in the MPP group and 72% in the DPP group would experience new mood episodes, and considering a two-sided alpha of 5% and 80% statistical power, the estimated sample size required was 15 participants per group.

## Results

**Table 1** summarizes the sociodemographic and clinical characteristics of the participants at baseline. This study included 22 patients in total, 4 men and 18 women, with a mean age of 41.3 (SD: 9.1). The sample was divided into two groups, according to their respective predominant polarities, 15 in MPP and 7 DPP, less than sample size estimation. There were no differences between groups for any of the demographic and clinical variables included in the study (**Table 1**).

**Table 1:** Demographic and clinical variables of MPP and DPP subjects.

Characteristics	MPP (n=15)	DPP (n=7)	P-value
Age (years)*	40.93 (7.93)	40.86 (12.54)	0.986
Gender (% male)**	20.0	14.3	0.746
Type of BD (type 1)**	13 (86.7)	4 (57.1)	0.124
Age of onset (years)*	21.27 (7.57)	23.14 (11.408)	0.650
Time since onset of illness*	10.50 (8.73)	10.27 (5.96)	0.977
Number of Hospitalizations*	1.53 (2.35)	0.14 (0.378)	0.141
Psychotic Symptoms**	6 (40.0)	3 (42.9)	0.899
Eating Disorders**	1 (6.7)	2 (28.6)	0.163
Anxiety Disorders**	6 (40.0)	3 (42.9)	0.899
Substance Abuse**	2 (13.3)	1 (14.3)	0.952

**Figure 1:**
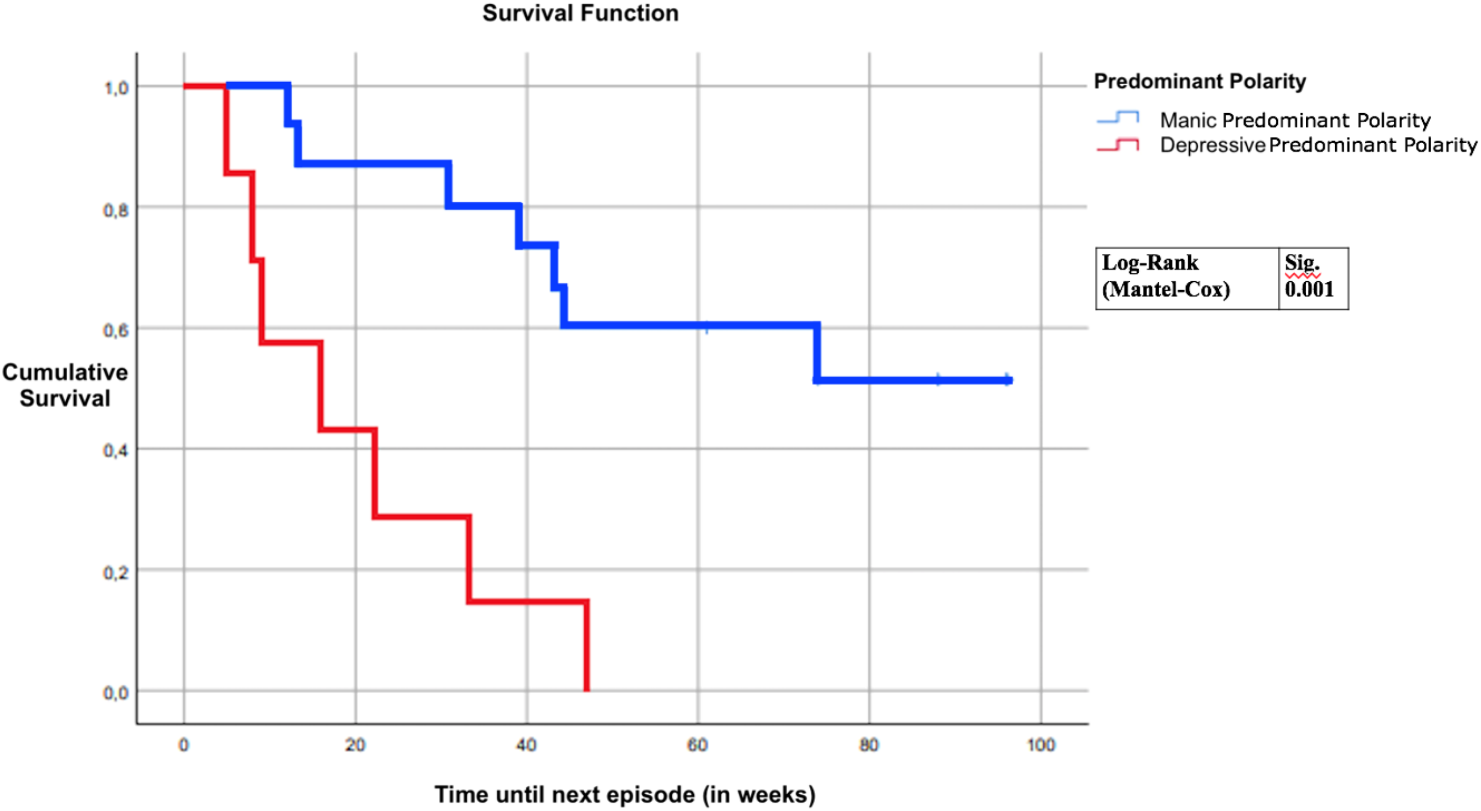
Predominant polarity vs time until next episode.

The proportion of individuals with new episodes during the follow-up period was 100% in DPP and 46.7% in MPP groups. The confidence interval for the average time to first episode was 36.57 ± 15.69 = (20.88, 52.26) for MPP and 20 ± 11.8527 = (8.1473, 31.853) for DPP. Regarding our main hypothesis, a log-rank survival analysis (Kaplan-Meyer) revealed that MPP patients remained in remission for longer periods of time in comparison to DPP patients (Log Rank 11.832; p = 0.001) (**Figure 1**). The median time to relapse was 39 weeks for MPP and 16 for DPP.

We also have investigated the usage of medications between groups following rational from previous studies (32). In these analyses, time until the first pharmacological modification is computed in a survival curve. Considering the challenges of long-term BD treatment, it is highly desirable to minimize medication changes. Furthermore, the need for dose and medication changes is a direct measure of an individual’s clinical stability. Therefore, we complementarily analyzed the time to the first medication change for each type of medication used by the patients in the study: antidepressants, antipsychotics, lithium and others mood stabilizers. These analyses showed that the MPP group remained longer without changing antidepressants when compared to the DPP group (Log Rank 8.246; p = 0.004). No other class of medications showed a statistically significant difference between the groups.

## Discussion

To the best of our knowledge, this is the first study to investigate the role of PP in the response to psychotherapy in subjects with BD. In accordance with our exploratory hypothesis, patients with different PP responded differently to CBT. A survival analysis revealed that MPP patients remained episode-free for longer after receiving CGBT treatment, in comparison to DPP patients, about 16 weeks more. At a first glance, our findings were counterintuitive since previous literature suggested that in comparison to TAU CBT is associated with greater stabilization of residual depressive symptoms, frequently observed in DPP, as well as prevention of new depressive episodes (13). One possible explanation for these findings is the greater presence of BD type I in the MPP group (87%) in comparison to DPP (57%), although this difference was not statistically significant. Previous meta-analyses have already reported better response to psychotherapeutic treatments in BD type I when compared to type 2 (14, 17). Such an argument would be in line with studies that indicate that BD type 2 is associated with a greater number of depressive symptoms, cognitive complaints, greater functional impairment, and the presence of personality disorders (33).

Another possible explanation is that symptoms of manic episodes are easier to identify at the beginning of its presentation, in comparison to depressive episodes; thus, making it easier to spot early episode signs and prevent it from becoming a full-blown manic episode. The opposite is also true regarding depressive episodes, which often evolves more gradually, making early recognition more difficult. The individuals in the study were encouraged to monitor their mood daily, using a mood graph, which may have facilitated this discrepancy. Another aspect of our CBT protocol that may have influenced a better response in the MPP group is the creation of sleep daily routines. Sleep hygiene may interfere differently to depression and mania in bipolar disorder, though none previous trial has evaluated this hypothesis.

On the other hand, one could wonder why our intervention was not as effective for people with predominantly polar depression. One of the key elements in dealing with depressive episodes in bipolar and unipolar disorder is the identification and restructuring of cognitive distortions (13, 34) and although our protocol dedicated a number of sessions comparable to others to addressing them, it may have been insufficient for DPP bipolar individuals. In addition to the duration of the protocol, another aspect that could be considered in the response to psychotherapeutic treatment in BD is the format used. The majority of recent studies with psychosocial approaches for BD have been developed to be conducted in group format (35, 36). Considering the high costs associated with BD (37) and the urgency these patients require to be treated (38), this decision is understandable and desirable. However, group psychotherapies have some limitations, such as time per person talking during sessions. No study to date has directly compared the effect of group therapy versus individual therapy on BD. However, a recent meta-analysis has shown that psychoeducation, a core component in several structured psychotherapy approaches for BD, is more effective in reducing recurrences when delivered in group than individual format (13). Unfortunately, as these authors suggested, it was uncertain if CBT techniques works better in individual or in group format. Future clinical trials with BD should investigate this literature gap in association with PP.

## Limitations

This study has several limitations that should be acknowledged. First, the relatively small sample size may have limited the statistical power to detect subgroup effects. Second, the absence of participants classified as having undetermined or mixed polarity may restrict the generalizability of the findings, given that such clinical presentations are commonly reported in bipolar disorder. Additionally, mixed affective states were not included in the operational definition of predominant polarity, which may have resulted in an oversimplification of the illness course. Future studies should consider incorporating mixed states and longer follow-up periods to enhance the precision and clinical applicability of polarity-based classifications. The choice of telephone follow-up interviews ensured adequate frequency of assessments; however, it should be acknowledged as a potential source of bias since it limits the behavioral assessment of patients, especially during episodes of depression.

This study is a *post-hoc* exploratory analysis, which results in loss of the original randomization structure and increases the risk of bias. The small sample size, particularly within the Depressive Predominant Polarity subgroup, limits statistical power and generalizability. No multivariable adjustment was performed to account for potential clinical or sociodemographic confounders, which may have influenced the results. The absence of a prospectively registered protocol further constrains methodological transparency. Consequently, the findings should be interpreted as exploratory and hypothesis-generating, requiring confirmation in larger, prospectively designed studies.

## Conclusion

The current study shows that MPP BD patients remained symptom-free for a longer duration than DPP patients (Log Rank = 11.832, p = 0.001), indicating a more favorable response for MPP after CBGT sessions, suggesting that the PP specifier may be an important moderator of CBGT response in BD. This has relevant clinical implications when prescribing CBT as maintenance add-on treatment for BD. Future studies in subjects with BD should include larger sample groups to assess the possible influence of the PP specifier in CBT and other types of maintenance psychotherapy.

## Data Availability

The raw data supporting the conclusions of this article will be made available by the authors, without undue reservation.
